# Dual roles of [CHCA + Na/K/Cs]^+^ as a cation adduct or a protonated salt for analyte ionization in matrix‐assisted laser desorption/ionization mass spectrometry

**DOI:** 10.1002/rcm.9111

**Published:** 2021-05-27

**Authors:** Xianwen Lou, Galen Miley, Joost L. J. Van Dongen

**Affiliations:** ^1^ Laboratories of Macromolecular and Organic Chemistry Eindhoven University of Technology P.O. Box 513 Eindhoven MB 5600 The Netherlands; ^2^ Laboratory of Chemical Biology, Department of Biomedical Engineering Eindhoven University of Technology P.O. Box 513 Eindhoven MB 5600 The Netherlands

In matrix‐assisted laser desorption/ionization mass spectrometry (MALDI MS), matrix ions are generated by laser irradiation, and the secondary charge transfer reactions between these matrix ions and analyte molecules are essential for the ionization of analytes.^1^, ^2^, ^3^, ^4^, ^5^ α‐Cyano‐4‐hydroxycinnamic acid (CHCA) is one of the most commonly used matrices due to its ability to efficiently generate analyte ions for various types of compounds. When CHCA is used as a matrix, protonated ions and alkali metal ion adducts are normally observed in the positive ion mode. Accurate interpretation and assignment of the various types of matrix ions are crucial to understand how MALDI ions are formed and how subsequent ionization of target analytes proceeds. Although [CHCA + Na/K]^+^ is usually assigned as sodiated/potassiated matrix molecules, we demonstrate that these ions are not simply alkali metal ion adducts. We show that they can be transformed into protonated ions of the corresponding matrix salts. The interconversion of these matrix ions has yet to be seriously interrogated thus far.

Figure 1 shows a typical MALDI TOF MS spectrum of CHCA. The major peak at *m/z* 212.0 is [CHCA + Na]^+^, and is usually assigned as the sodium ion adduct of CHCA. Because of the ubiquitous presence of sodium impurities, addition of extra salts is generally not required for the formation of the alkali metal ion adduct. Most likely, [CHCA + Na]^+^ is formed in the gas phase by the adduction of Na^+^ to the matrix molecule, and, therefore, [CHCA + Na]^+^ is intuitively called the Na^+^ adduct of the matrix.^2^, ^3^, ^4^ However, by considering the molecular structure, it is also possible that this sodiated molecule can be transformed into a protonated ion of CHCA sodium salt (see Scheme 1).

**FIGURE 1:**
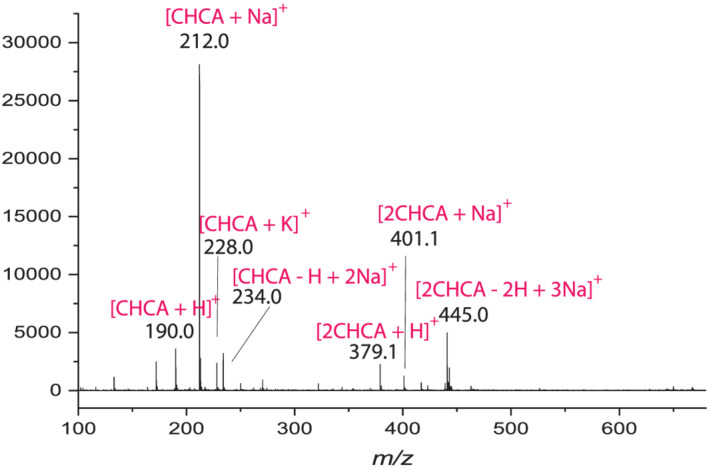
A typical MALDI TOF MS spectrum of CHCA

**SCHEME 1:**
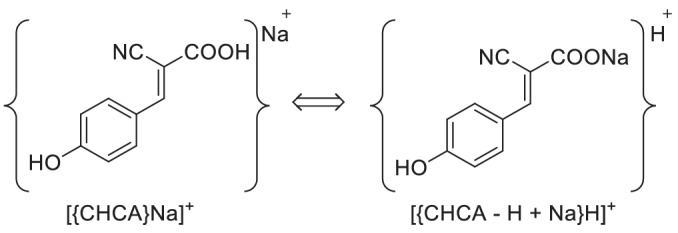
Transformation between [(CHCA)Na]^+^ and [{CHCA − H + Na}H]^+^

In Scheme 1 [{CHCA}Na]^+^ denotes the sodiated matrix molecule, and [{CHCA − H + Na}H]^+^ represents the protonated ion of CHCA sodium salt. Based on this reaction, [CHCA + Na]^+^ can swap between alkali metal ion adducts and protonated ions. This interchange reaction is extremely important to a full understanding of the secondary charge transfer reactions between matrix ions and analyte molecules in MALDI. For the ionization of analyte molecules, sodiated matrix molecules can provide Na^+^ while protonated ones can provide H^+^. We demonstrated that [CHCA + Na]^+^ can yield an alkali metal ion as [{CHCA}Na]^+^ or yield a proton as [{CHCA − H + Na}H]^+^ to an analyte molecule depending on the properties of the analytes.

The MALDI TOF MS measurements were performed with an Autoflex Speed instrument (Bruker, Bremen, Germany) equipped with a 355 nm Nd:YAG smartbeam laser with maximum repetition rate of 1000 Hz, capable of executing both linear and reflector modes. The accelerating voltage was held at 19 kV and the delay time at 130 ns for all experiments. Mass spectra were acquired in the reflector positive ion mode by summing spectra from 500 random laser shots at an acquisition rate of 100 Hz. Matrix solutions were freshly prepared in tetrahydrofuran (THF) at a concentration of approximately 20 mg/mL. Sodium trifluoroacetate (NaTFA), potassium trifluoroacetate (KTFA) and CsI_3_ were also dissolved in THF at approximately 20 mg/mL. Polyethylene glycol (PEG with OH and H end groups, average molecular weight 600) and didecylamine (DDA) were selected as the analytes for the alkali metal ion transfer and the proton transfer reactions, respectively.

It is relatively straightforward to determine whether a matrix ion is a Na^+^ adduct. As a cation adduct, Na^+^ will be stripped from the matrix as compounds with higher Na^+^ affinity are introduced, resulting in suppression of the sodiated matrix molecule. PEGs are well‐known to have a high Na^+^ affinity. Figure 2 shows a MALDI TOF MS spectrum of PEG‐600 with the CHCA matrix. Indeed, high PEG peaks were observed at the expense of [CHCA + Na]^+^. The charge transfer reaction can be described by Scheme 2.(2)${\left[\left\{\text{CHCA}\right\}\mathrm{Na}\right]}^{+}+\mathrm{PEG}\Rightarrow \left\{\text{CHCA}\right\}+{\left[\mathrm{PEG}+\mathrm{Na}\right]}^{+}$Following this scheme, [CHCA + Na]^+^ is acting as a Na^+^ adduct. It can be seen in Figure 2 that the [CHCA + Na]^+^ peak was completely suppressed by PEG‐600. However, it is interesting to see that the [CHCA + H]^+^ peak, which was significantly smaller than the [CHCA + Na]^+^ peak when CHCA was measured alone (see Figure 1), can still be observed. Apparently, PEG can only remove Na^+^ from [{CHCA}Na]^+^ but not H^+^ from [{CHCA}H]^+^. The results shown in Figure 2 clearly indicate that [CHCA + Na]^+^ can deliver Na^+^ as a sodiated molecule of [{CHCA}Na]^+^.

**FIGURE 2:**
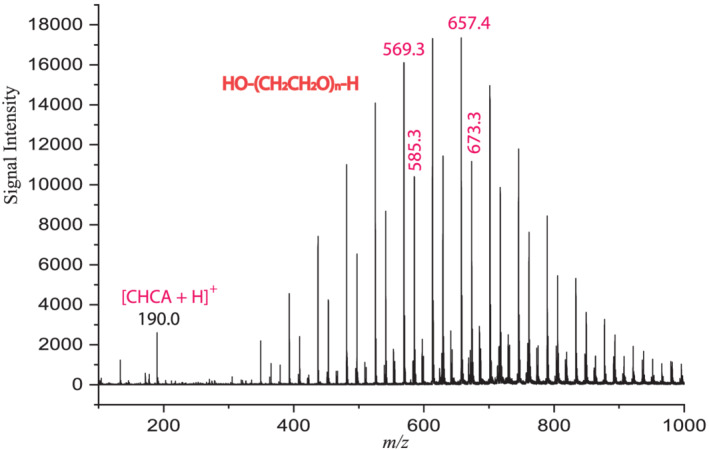
MALDI TOF MS spectrum of PEG using CHCA matrix showing [CHCA + Na]^+^ acting as [{CHCA}Na]^+^. CHCA and PEG‐600 were dissolved in THF and mixed at a mole ratio of CHCA/PEG 10:1

In order to prove that [CHCA + Na]^+^ can also provide a proton as a protonated matrix ion of [{CHCA − H + Na}H]^+^, DDA was chosen as the analyte because it can easily be ionized by protonation. For an unequivocal validation of the assumption of proton transfer from [{CHCA − H + Na} + H]^+^ to DDA, the following two conditions must be met, (1) [{CHCA − H + Na} + H]^+^ is the solitary source of protons for the gas‐phase proton transfer reaction, and (2) DDA is only protonated by a gas‐phase proton transfer reaction. To satisfy these two requirements, efforts were made to ensure that ionization of DDA other than protonation via proton transfer from [{CHCA − H + Na}H]^+^ in the gas phase is avoided. The protonated matrix ions, [{CHCA}H]^+^ and [{CHCA}_2_H]^+^, can be other major sources of protons in secondary proton transfer reactions. Therefore, experiments were designed to completely suppress the formation of these ions, while still yielding abundant [CHCA + Na]^+^ ions. By mixing CHCA with a suitable amount of NaTFA, the protonated matrix ions can be suppressed entirely, as is seen in Figure 3A. The main observed peaks are [CHCA + Na]^+^ at *m/z* of 212.0, [{CHCA − H + Na}Na]^+^ at 234.0, [{CHCA − H + Na}_2_Na]^+^ at 445.0, and [{CHCA − H + Na}_3_Na]^+^ at 656.1. As for minimizing the chance of DDA protonation by the acidic matrix prior to laser irradiation, mass spectra were acquired on the spot of CHCA mixed with NaTFA in the presence of DDA vapor. DDA vapor was provided by loading a small amount of DDA near the matrix spot. In the loading of DDA, care was taken to prevent any direct contact of DDA with the matrix spot, preventing non‐gas‐phase protonation. The MALDI TOF MS spectrum obtained is shown in Figure 3B. Clearly, abundant [DDA + H]^+^ ions were detected. We argue that the ionization reaction for DDA can be described by Scheme 3.

(3) $${\left[\left\{\text{CHCA}-H+\mathrm{Na}\right\}H\right]}^{+}+\mathrm{DDA}\Rightarrow \left\{\text{CHCA}-H+\mathrm{Na}\right\}+{\left[\mathrm{DDA}+H\right]}^{+}$$

Similar results were also obtained when the experiments were repeated with KTFA instead of NaTFA.

**FIGURE 3:**
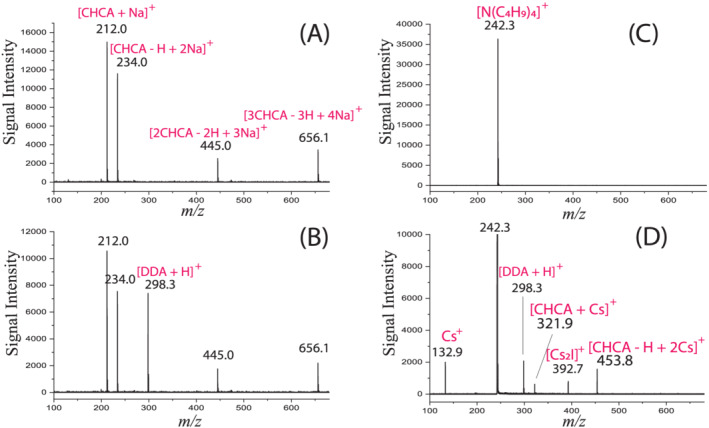
MALDI TOF MS spectra of didecylamine (DDA) vapor showing [CHCA + Na/Cs]^+^ acting as [{CHCA − H + Na/Cs}H]^+^: A, CHCA + NaTFA (the mole ratio of CHCA/NaTFA is 8:1) in the absence of DDA vapor; B, DDA deposited near the spot of CHCA + NaTFA (the mole ratio of CHCA/NaTFA is 8:1); C, DDA deposited near the spot of CHCA + TBAI (the mole ratio of CHCA/TBAI is 100:1); and D, DDA deposited near the spot of CHCA + TBAI + CsI_3_ (the mole ratio of CHCA/CsI_3_/TBAI is 100/50/1)

In another set of experiments, tetrabutylammonium iodide (TBAI) was used to suppress the formation of protonated matrix ions. It has been reported that TBAI can suppress the matrix ions very effectively.^6^, ^7^ As expected, no protonated DDA ions were detected out of the DDA vapor because all reactive matrix ions were sequestered by TBA (Figure 3C). Interestingly, [DDA + H]^+^ ions recovered when a large amount of CsI_3_ was added to the CHCA and TBAI mixture (Figure 3D). Cs^+^ cannot ionize DDA directly, therefore protonation of DDA must be due to reactions caused by the addition of CsI_3_. When an excessive amount of Cs^+^ is added, TBA cannot sequester the release of Cs^+^ completely. The escaped Cs^+^ reacts with matrix molecules in the gas phase forming [{CHCA}Cs]^+^ which will then be transformed to [{CHCA − H + Cs}H]^+^. Here, we argue that [{CHCA − H + Cs}H]^+^ ions are operative in the protonation of DDA molecules in the MALDI gas‐phase plume. The results strongly support the assumption of the interconversion between [{CHCA}Cs]^+^ and [{CHCA − H + Cs}H]^+^. In addition to CHCA, similar results were obtained with 2,5‐dihydroxybenzoic acid (DHB), another commonly used matrix bearing similar molecular functionality (see Figure 4).

**FIGURE 4:**
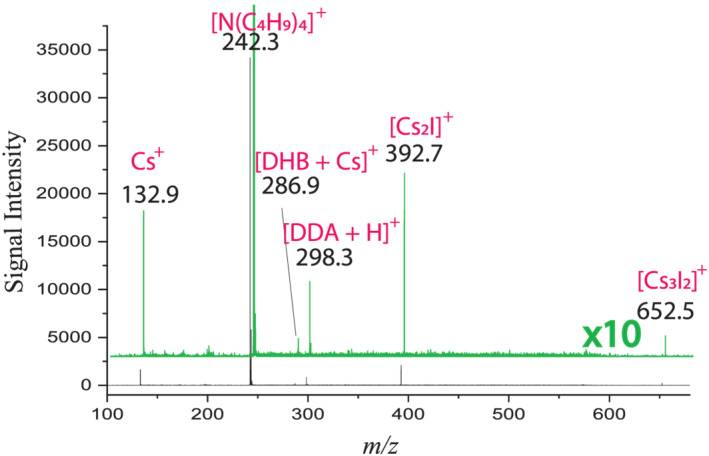
MALDI TOF MS spectra of didecylamine (DDA) vapor with DHB matrix showing [DHB + Cs]^+^ acting as [{DHB − H + Cs}H]^+^. DDA was deposited near the spot of BTAI + CsI_3_ + DHB (the mole ratio of TBAI/CsI_3_/DHB is 1/100/200)

In conclusion, we have shown that CHCA matrix can interconvert between alkali metal ion adducts and protonated ions of their corresponding salts. Understanding the circumstances around this interconversion is important in rationalizing the formation of the final analyte MALDI ions recorded in the mass spectra. As the [matrix + Na/K]^+^ ions are among the most frequently observed matrix ions for many matrices, we should be aware that these ions can swap between alkali metal ion adducts [{matrix}Na/K]^+^ and protonated salt ions [{matrix − H + Na/K}H]^+^. It is hoped that the results presented provide a more nuanced understanding of the ionization mechanics at work in the MALDI gas‐phase plume.

### PEER REVIEW

The peer review history for this article is available at https://publons.com/publon/10.1002/rcm.9111.

## Data Availability

Data available on request from the authors.
